# *Culex quinquefasciatus* Density Associated with Socioenvironmental Conditions in a Municipality with Indeterminate Transmission of Lymphatic Filariasis in Northeastern Brazil

**DOI:** 10.3390/pathogens13110985

**Published:** 2024-11-11

**Authors:** Amanda Xavier, Cristine Bonfim, Pablo Cantalice, Walter Barbosa Júnior, Filipe Santana da Silva, Vítor Régis, André Sá, Zulma Medeiros

**Affiliations:** 1 Graduate Program in Health Sciences, University of Pernambuco, Recife 50100-010, Brazil; zulma.medeiros@fiocruz.br; 2 Department of Parasitology, Oswaldo Cruz Foundation, Aggeu Magalhães Institute, Recife 50670-420, Brazil; walter.lins@fiocruz.br (W.B.J.); vitor.regis@upe.br (V.R.); 3 Department of Nursing, Vitória de Santo Antão Academic Center, Federal University of Pernambuco, Vitória de Santo Antão 55608-680, Brazil; 4 Social Research Directorate, Joaquim Nabuco Foundation, Ministry of Education, Recife 52061-540, Brazil; cristine.bonfim@uol.com.br; 5 Graduate Program in Public Health, Federal University of Pernambuco, Recife 50670-901, Brazil; 6 Graduate Program in Public Health, Oswaldo Cruz Foundation, Aggeu Magalhães Institute, Recife 50670-420, Brazil; 7 Department of Genetics, Federal University of Pernambuco, Recife 50740-600, Brazil; cantalice.pablo@gmail.com; 8 Department of Biomedical Engineering, Center for Technology and Geosciences, Federal University of Pernambuco, Recife 50670-901, Brazil; filipe@ufcspa.edu.br; 9 Faculty of Medical Sciences, University of Pernambuco, Recife 50100-130, Brazil; 10 Statistics and Geoprocessing Center, Aggeu Magalhães Institute, Recife 50670-420, Brazil; andre.sa@cpqam.fiocruz.br

**Keywords:** lymphatic filariasis, *Culex quinquefasciatus*, vector density index, social deprivation indicator

## Abstract

Lymphatic filariasis (LF) is a neglected tropical disease associated with poverty and poor environmental conditions. With the inclusion of vector control activities in LF surveillance actions, there is a need to develop simple methods to identify areas with higher mosquito density and thus a higher consequent risk of *W. bancrofti* transmission. An ecological study was conducted in Igarassu, which is in the metropolitan region of Recife, Pernambuco, Brazil. The mosquitoes were captured in 2060 houses distributed across 117 census tracts. The vector density index (VDI), which measures the average number of lymphatic-filariasis-transmitting mosquitoes per number of houses collected in the risk stratum, was constructed. Moreover, the social deprivation indicator (SDI) was constructed and calculated through principal component factor analysis. An average of 242 female *C. quinquefasciatus* were found in the high-risk stratum, while the average in the low-risk stratum was 108. The overall VDI was 6.8 mosquitoes per household. The VDI for the high-risk stratum was 13.2 mosquitoes per household, while for the low/medium-risk stratum, it was 5.2. This study offers an SDI for the density of *C. quinquefasciatus* mosquitoes, which can help reduce the costs associated with data collection and allows for identifying priority areas for vector control actions.

## 1. Introduction

Lymphatic filariasis (LF) is a neglected tropical disease transmitted by mosquitoes and associated with poverty and poor environmental conditions [1]. The Global Programme to Eliminate Lymphatic Filariasis (GPELF) was launched in 2000 by the World Health Organization (WHO) with the goal of eliminating the disease as a public health problem by 2030 [2].

In Brazil, LF is an urban disease, and its etiological agent is *Wuchereria bancrofti*, with *Culex quinquefasciatus* being the transmitting mosquito [3]. Maceió-Alagoas [4], Belém–Pará [5], and Manaus–Amazonas [6] have identified the indicators of transmission interruption. In Pernambuco, Recife, Olinda, and Jaboatão dos Guararapes are under surveillance after mass drug administration (MDA), while Paulista provides individual treatment for cases due to its low infection prevalence [7]. Nine other municipalities, Abreu e Lima, Cabo de Santo Agostinho, Camaragibe, Igarassu, Ilha de Itamaracá, Ipojuca, Itapissuma, Moreno, and São Lourenço da Mata, which are adjacent to endemic areas, are considered to have an undetermined transmission of filariasis [8].

These nine municipalities face serious environmental and social problems, such as inadequate water supply, a lack of basic sanitation, precarious housing conditions, low income, and high population density in the poorest areas [9]. This scenario favors the proliferation of mosquitoes, especially the species *C. quinquefasciatus*, intensifying the contact of these vectors with human populations and increasing the risk of disease transmission [10,11]. In the specific case of lymphatic filariasis (LF), the presence of the mosquito vector, associated with unplanned urbanization and migratory flows between municipalities, represents a high risk for the transmission of filarial infection [12,13].

The quantity and types of factors that influence the distribution of mosquitoes can vary significantly between different geographical regions. Thus, we must identify the local factors that determine the distribution and abundance of these vectors, facilitate the prediction of disease transmission cycles, and implement surveillance and intervention measures targeted to the real needs of small areas [10]. Composite indices based on environmental and socioeconomic factors related to the *C. quinquefasciatus* vector can be useful tools for this identification [13,14,15].

This study addressed a public health concern, particularly in regions facing ongoing challenges related to LF. Focusing on mosquito density and socioenvironmental factors is timely and essential for guiding control measures. With the inclusion of vector control activities in LF surveillance actions, there is a need to develop simple methods of identifying areas with higher mosquito density and thus a higher consequent risk of *W. bancrofti* transmission [16,17]. From this perspective, the hypothesis in this study was that greater socioenvironmental deprivation leads to an increase in mosquito density, thus increasing the risk of LF transmission. The objective of this study was to analyze the spatial distribution of *C. quinquefasciatus* density according to the socioenvironmental conditions in an area with an undetermined transmission of *W. bancrofti*.

## 2. Materials and Methods

This study was conducted in the municipality of Igarassu, located in the metropolitan region of Recife, Pernambuco, northeastern Brazil. It is one of the nine municipalities with an undetermined LF status in Pernambuco, northeastern Brazil [8]. Historically, it has had cases of microfilaremia [18] identified and no treatment interventions in its population. Furthermore, there is no record of any investigation into *C. quinquefasciatus* mosquitoes. An ecological study was conducted, with the unit of analysis being the urban census tracts of Igarassu. An ecological study was conducted using the 131 urban census sectors of Igarassu as the unit of analysis. Of these, 14 census sectors were excluded from the analysis due to a lack of available information.

The mosquitoes were captured in 2060 houses distributed across 117 census tracts. Maps of the census tracts from the IBGE website [9] were consulted and then manipulated to define quadrants. In each quadrant, a line was drawn diagonally across the 2nd and 3rd quadrants, connecting opposite quadrants. From the midpoint of each quadrant (2nd and 3rd), the streets were defined, in this case, two, that were in each midpoint of these quadrants. For each street, 10 households were selected, totaling 20 per census tract. This selection followed the listing of the first 10 households (according to the numbering) located on the right side of the street.

The heads of households were given an informed consent form, and, upon their agreement to participate, signed the form. Collections were then conducted once in each household in March 2019 and September 2019 between the hours of 9 and 12 AM using electric aspirators indoors, following the protocol by Ramesh et al. [19]. The mosquitoes were aspirated and stored in fine mesh cages for later storage in a −20 °C freezer. The mosquitoes were identified based on the characteristics described by Forattini et al. (1965) [20].

The vector density index (VDI) measures the average number of lymphatic-filariasis-transmitting mosquitoes per number of houses collected in the risk stratum. It was calculated using the following formula:

$$\mathrm{VDI}=\frac{number\;of\;female\;Cx.quinquefasciatus\;captured\;per\;risk\;stratum}{number\;of\;households\;analyzed\;per\;risk\;stratum}$$

For the construction of the social deprivation indicator (SDI), the Pearson correlation of 10 socioenvironmental variables provided by the 2010 demographic census (Table 1) was measured, and only variables that showed statistical significance (*p* < 0.05) remained in the process. These variables were selected because they collectively represent situations of social deprivation and reflect information associated in the literature with the breeding sites and the proliferation of *C. quinquefasciatus* mosquitoes [12,14].

Before the estimation of the SDI, the variables composing it were examined using the Kaiser–Meyer–Olkin (KMO) [21] test and Bartlett’s test of sphericity [22].

The correlation between the eligible variables and the number of female mosquitoes was evaluated using Spearman’s correlation [23]. Variables with a significant correlation were selected. The selected variables were then normalized to belong to a 0–1 interval using the following equation:

$$Z_{i}=\frac{X_{i}-min\left(X\right)}{max\left(X\right)-min\left(X\right)}\;$$

where

*i* = census tract;
*X* = variable to be normalized.

After finding the acceptable factors, the index was normalized to the 0–1 range using

$$SDI=\frac{CP_{i}-min\left(CP_{i}\right)}{max\left(CP_{i}\right)-min\left(CP_{i}\right)}\;$$

The construction of the SDI was carried out through principal component factor analysis, which reduces many variables to a smaller number, now referred to as factors. The variables forming a factor need to be correlated with each other for the model to be appropriate [24]. The technique produces regression coefficients (loadings or factorial loadings) that indicate the relationship between the factor and each original variable. Additionally, it determines the percentage of total variance explained for each extracted factor. In this study, among the extracted factors, the one that explained the most variance (the first factor) was selected. This factor constituted the SDI [25].

Factor analysis is useful for identifying latent (unobservable) factors from a set of variables (usually correlated with each other). This strategy is used when the type of information being studied is difficult (or impossible) to measure. In our case, various variables on social deprivation were used to estimate a latent factor representing this information, increasing the accuracy of the estimate (as it involves several variables with different weights) with the least possible loss of information. This technique was selected to construct the SDI because it allowed the chosen variables to be those that were most correlated with the number of mosquitoes.

The correlation between the variables that make up the SDI was not an issue, as PCA eliminated this aspect of the analysis. Moreover, the correlation between variables was a prerequisite for applying PCA, as indicated by the KMO index. To obtain strata, the SDI was subjected to the k-means clustering technique, in which the number of SDI bands was identified by an elbow graph. To explain the relationship between the SDI and the VDI, the Poisson inverse Gaussian (PIG) regression model [26] was employed, which showed adjustment according to the generalized Akaike metric (GAIC) [27].

The PIG model was chosen because it better fit the data. For this verification, the GAIC (generalized Akaike information criterion) was computed for each of the cited distributions, with the one having the lowest GAIC (best fit) being selected in the end. Before the modeling with the PIG distribution, a step was conducted to test other, more traditional distributions. The distributions tested and the respective results were as follows: Poisson (GAIC = 18,907.5), negative binomial (GAIC = 1223.5) and Poisson inverse Gaussian (GAIC = 1198.6). All calculations were performed using the R statistical programming language version 4.1.0, and the adopted significance level was 5%.

The overdispersion of the model was not evaluated for the different SDI strata, meaning that both strata presented the same overdispersion. This means that although the variance was not necessarily equal, the distance of the variance from the mean of each stratum was the same. Thus, the stratum with the higher mean had a greater variance than the stratum with the lower mean, implying the need to consider other factors when determining the number of mosquitoes for higher SDI strata.

This study was approved by the Research Ethics Committee of the Instituto Aggeu Magalhães, FIOCRUZ-PE, under approval number 039627/2019.

## 3. Results

The seven variables that comprised the SDI (*p* < 0.05) were the proportion of households without public water supply, proportion of households without adequate sewage systems, proportion of households without garbage collected by sanitation services, proportion of households with six or more residents, proportion of illiterate household heads, per capita household income, and per capita income of household heads.

Bartlett’s test of sphericity (χ^2^ = 372.47; *p* < 0.01) and the KMO (0.68) indicated that the correlations among the variables were suitable for exploratory factor analysis. There was a statistically significant correlation among the seven eligible variables for forming the SDI, considering Spearman’s correlation (Table 2). The variables with the greatest weight in the index were per capita household income (−0.474), the proportion of illiterate household heads (0.466), and per capita income of household heads (0.466).

Table 3 presents the correlation matrix, applying exploratory factor analysis via principal components. The index is represented according to Equation (2); 43.7% of the general variance is explained by component 1, which constitutes the SDI:

$$SDI=0.191X_{1}+0.261X_{2}+0.332X_{3}+0.388X_{4}+0.466X_{5}+\left(-0.474X_{6}\right)+\left(-0.439X_{7}\right)$$

*X*_1_ is the proportion of households without public water supply;
*X*_2_ is the proportion of households without adequate sewage system;
*X*_3_ is the proportion of households without garbage collected by sanitation services;
*X*_4_ is the proportion of households with six or more residents;
*X*_5_ is the proportion of illiterate household heads;
*X*_6_ is the per capita household income;
*X*_7_ is per capita income of household heads;

**Table 3:** Correlation matrix of variables regarding socioenvironmental conditions by census tract, Igarassu, 2022.

KMO (0.68)	Variable	WATS	ISEW	GARB	HOUS	ILIND	RENDo	RENp
0.56	WATS	1	0.01	0.23	0.27	0.46	−0.05	0.06
0.60	ISEW		1	0.10	0.49	0.24	−0.24	−0.20
0.87	GARB			1	0.25	0.47	−0.36	−0.30
0.68	HOUS				1	0.54	−0.32	−0.30
0.75	ILIND					1	−0.53	−0.53
0.59	RENDo						1	0.93
0.57	RENp							1

**Figure 1:**
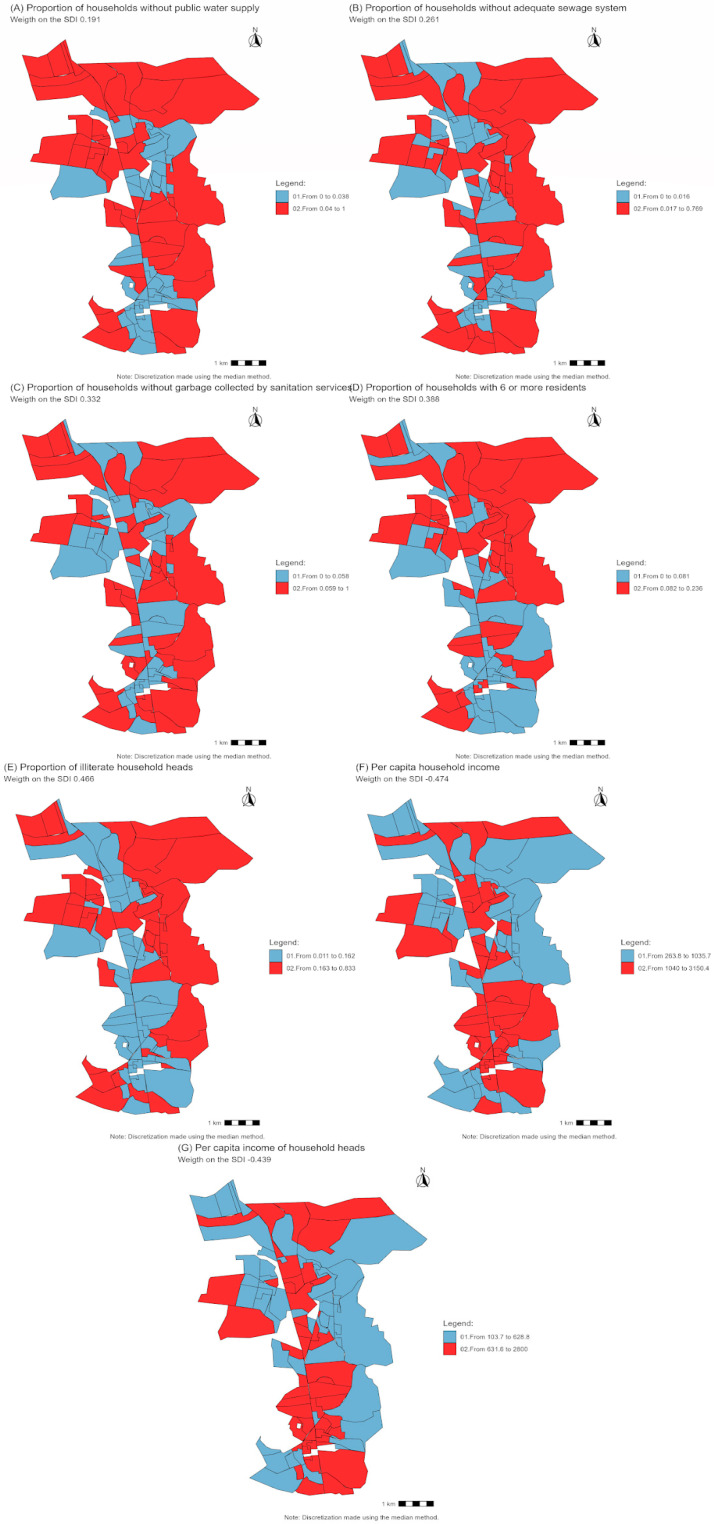
Spatial representation of the variables used in the social deprivation indicator, Igarassu, 2022.

To create the SDI strata, the k-means clustering technique was applied, resulting in four chosen strata. The results for the four strata are presented in Table 4. Strata 1, 2, and 3 (very low risk, low risk, and medium risk) were not statistically significant. Stratum 4 (high risk) was statistically significant, meaning that the average number of female *C. quinquefasciatus* in this stratum was statistically different from the others. Therefore, it was useful to merge the strata that were not significant. Figure 1 shows the distribution of the variables that make up the SDI, highlighting the differences between the census tracts. The red areas indicate regions with greater deprivation or lower quality in the aspects analyzed, while the blue areas represent locations with better conditions for the variable in question.

The average number of female *C. quinquefasciatus* found in the high-risk stratum was 242, while the low-risk stratum had an average of 108. This means the high-risk stratum had 2.24 times more mosquitoes on average than the low-risk stratum (*p* < 0.01, Table 4).

**Figure 2:**
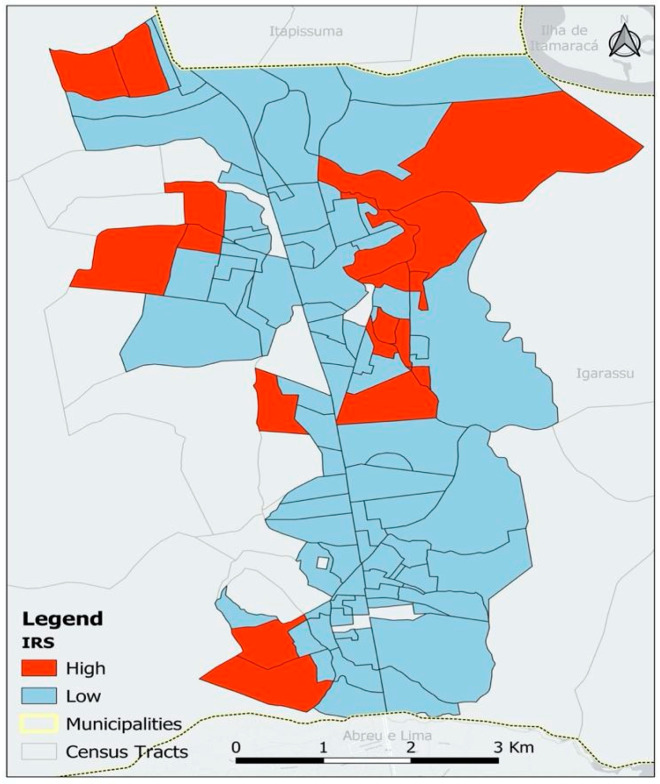
Spatial representation of the social deprivation indicator, Igarassu, 2022.

A total of 26,027 *C. quinquefasciatus* were collected from the 2060 investigated households. Of the captured mosquitoes, 14,920 (58%) were female; among them, 8783 (59%) were engorged. The overall vector density index (VDI) was 6.8 mosquitoes per household. Figure 2 shows the distribution of the SDI by census tract. The VDI for the high-risk stratum was 13.2 mosquitoes per household, while for the low/medium-risk stratum, it was 5.2 (*p* < 0.01).

## 4. Discussion

Due to the complexity of lymphatic filariasis (LF), the environment, and vectors, strategies for its elimination as a public health problem can be tailored to environmental and socioeconomic specifics [28,29]. Stratifying space according to these factors serves as a supporting tool for planning disease control actions [12,17,30].

The SDI proposed in this study was constructed from variables that reflect the socioenvironmental factors associated with the density of female *C. quinquefasciatus*. The worst socioenvironmental conditions were associated with a higher vector density index (VDI). The high-risk stratum consisted of 21 census tracts, 3 of which were considered high-priority areas for potential surveillance actions in the municipality.

The use of census tracts as the spatial unit of analysis in the development of the SDI offers the advantage of representing the most disaggregated level of population and socioenvironmental data, likely ensuring better homogeneity among the population. The ability to conduct analyses in smaller areas facilitates the implementation of selective and specific actions for controlling endemic diseases [12,31]. In Brazil, the demographic census uses census tracts for registration control, with updates occurring every ten years [9].

Unplanned urban areas are characterized by inadequate sanitary facilities and the presence of populations living in poverty, conditions that favor the formation of vector breeding sites and the transmission of infectious diseases [10,11]. In this study, the variables used to analyze the urban census tracts were related to precarious sanitation conditions, income level, housing conditions, and population density. These variables are often associated with *C. quinquefasciatus* breeding sites, which are artificial reservoirs filled with water, containing organic matter and decomposing materials. They have a dirty appearance and are located near human dwellings [32,33].

Simonsen and Mwakitalu (2013) [12], in a review on filarial disease in urban environments, identified that the most consistent determinant in epidemiological investigations of LF is the environment, specifically in unplanned areas with poor sanitation. This condition is favorable for the reproduction of *C. quinquefasciatus*, leading to high biting rates, greater contact with humans, and increased potential for the transmission of filarial infection. Additionally, the high population density in the households, low income, and reduced levels of knowledge are interrelated characteristics that support transmission or hinder the prevention of urban LF.

The analysis of the collective of these variables from a single data point formed by an index has greater explanatory power and reflects the different levels of access to urban infrastructure. This allows for a better understanding of the condition of a small area [13]. To this end, two risk strata were formed to classify the census tracts as low or high risk, with the latter having the worst social and environmental conditions and the highest vector densities, thus deserving attention from surveillance for the control of urban LF.

The highest vector density index (VDI) was also identified in the high-risk stratum, with 13.2 mosquitoes per household, while the areas considered low in risk had approximately 5.2 mosquitoes per household. Lupenza et al. (2021) [34] emphasized that a high number of mosquitoes increases the bite rates for household occupants, thereby increasing the risk of LF infection. As such, the vector control efforts within the Global Program to Eliminate Lymphatic Filariasis (GPELF) should focus on environmental improvements.

Monitoring LF infection in populations through risk indicators is a simple, easy-to-apply, and low-cost tool for filariasis elimination programs in urban areas [15,34,35,36]. The ability to link this method’s construction with the vector and the sanitary conditions of an area allows for tracking the risk of filariasis transmission in nonendemic locations, with low prevalence, and in the context of elimination. Thus, this study presents a possible method for territorial surveillance based on the detection of new transmission foci independent of human cases.

In 2022, Xavier et al. (2022) [14] used a socioenvironmental risk indicator, previously validated by Bonfim et al. (2011) [37], to identify the risk of the human prevalence of lymphatic filariasis (LF), aiming to recognize areas with a higher risk for breeding sites and the proliferation of *C. quinquefasciatus.* Although having similar objectives, this study presents an innovative approach by constructing an indicator through principal component analysis and considering 10 variables related to precarious socioenvironmental conditions. This represents a more robust methodology. Both tools proved effective and can be replicated according to the available resources and the intended purpose of their use.

In practical terms, the use of the SDI can be a valuable tool for public management strategies, allowing for the identification of priority areas for intervention in the control of LF in a remote and low-cost manner. This approach enables the better allocation of resources to regions that genuinely need attention, scaling actions based on the risk of illness in the population. In high-risk areas, greater investments should be allocated to improve sanitary conditions and infrastructure, in addition to the distribution of repellents and insecticides. Furthermore, local primary care teams should be encouraged to engage with the population to raise awareness about vector protection measures, such as the use of insecticides, repellents, and protective clothing, as well as the elimination of breeding sites and regular inspections to identify new breeding grounds.

The limitations of this study include the fact that the database used to construct the SDI was published in 2010. Although the 2020 census had already been conducted, its data were not available during the preparation of this study. However, it is emphasized that the 2010 data have been widely used, recognized, and validated, providing greater consistency in comparisons with previous studies and better compatibility with other databases, which ensures the methodological coherence of the indicator.

Another limitation to be considered is the impact of the seasonal variation in mosquito density. One way to avoid bias in the results is that vector collection should occur during critical periods, ensuring a more representative sample of the vector’s behavior during times of higher risk of disease transmission. In this study, the collection of *C. quinquefasciatus* was conducted during the rainy season in Pernambuco, specifically between March and September 2019. During this phase, the high temperatures and increased water availability favor the development of larvae and the proliferation of mosquitoes, making the sample more representative and ensuring the greater accuracy of the SDI analyses.

## 5. Conclusions

This study presents a tool for the rapid and cost-effective detection of priority areas for the control of *C. quinquefasciatus* mosquitoes, thereby contributing to efforts to combat the transmission of LF. Because it is related to vector density, the proposed SDI offers an effective estimate of the chances of filarial infection transmission in an indirect manner, independent of the presence of infection or disease cases. This is of utmost importance in areas that are progressing in the process of eliminating LF but need monitoring strategies.

The results of this study offer an SDI for the density of *C. quinquefasciatus* mosquitoes, which reduces the cost associated with data collection and allows for indicating priority areas for vector control actions. In a context more centered on LF control, the SDI may enable more efficient interventions by directly impacting cost reduction by eliminating the need for data collection. Furthermore, it could direct public health investment to areas of greater risk, focusing efforts on infrastructure improvement and health education activities where they are most needed.

The adaptation and validation of the SDI in other regions with different socioenvironmental characteristics can ensure that the tool is applicable to various epidemiological and geographical realities, advancing LF control. Additionally, investigations that integrate environmental and climatic data may further enhance the accuracy of the index, and its use can also be expanded to other vector-borne infections by assessing variables associated with other diseases, such as Zika, Chikungunya, dengue, and Oropouche fever.

## Figures and Tables

**Table 1:** Variables eligible for composing the social deprivation indicator, Igarassu, 2022.

Variable	Definition	Indicator
WATS	Households with inadequate water supply	Proportion of households without internal water plumbing and without access to public water supply network relative to the total number of permanent private households
BATHR	Households without exclusive use bathrooms for residents	Proportion of households without showers or bathtubs and exclusive use of toilet facilities for household residents
GARB	Households with inadequate garbage collection	Proportion of households with garbage collection by public or private company services relative to the total number of permanent private households
ELEC	Households without electricity	Proportion of households without any type of electricity supply
HOUS	Households with 6 or more residents	Proportion of households with 6 or more residents
SEW	Households without sewage system	Proportion of households without drainage system for waste from the bathroom or toilet
ISEW	Households with inadequate sewage systems	Proportion of households without plumbing for waste from the bathroom or toilet, connected to a collection system that leads to a general drainage system in the area, region, or municipality, even if the system does not have a sewage treatment plant
ILIND	Illiterate individuals who are household heads	Proportion of household heads who either did not know how to read and write, those who learned but forgot due to an unconsolidated literacy process, and those who could only sign their own name
RACE	Resident individuals self-declared as Black race or ethnicity	Proportion of resident individuals self-declared as Black race or ethnicity
REND	Individuals responsible with no positive income	Proportion of individuals responsible for permanent private households with no positive income, meaning no type of earnings in value

Source: IBGE, 2010 [9].

**Table 2:** Pearson and Spearman correlation between the variables comprising the SDI and the number of female *C. quinquefasciatus* mosquitoes in Igarassu, 2022.

Variable	Abbreviation	Pearson Correlation		Spearman Correlation	
		Estimate	*p*-Value	Estimate	*p*-Value
Proportion of households without public water supply	WATS	0.34	0.00	0.41	0.00
Proportion of households without exclusive-use bathrooms for residents	BATHR	0.06	0.54	0.34	0.00
Proportion of households without any kind of sewage system	SEW	0.06	0.54	−0.13	0.21
Proportion of households without adequate sewage system	ISEW	0.10	0.30	0.10	0.30
Proportion of households without garbage collected by sanitation services	GARB	0.37	0.00	0.32	0.00
Proportion of households without electricity	ELEC	0.03	0.74	0.23	0.02
Proportion of households with 6 or more residents	HOUS	0.16	0.11	0.22	0.03
Proportion of illiterate individuals responsible for the household	ILIND	0.26	0.01	0.10	0.30
Proportion of resident individuals of Black race/color	RACE	0.02	0.80	0.08	0.40
Proportion of household heads with no positive income	REND	−0.10	0.31	0.09	0.37
Per capita household income	RENDo	−0.21	0.03	−0.34	0.00
Per capita income of household heads	RENDp	−0.16	0.11	−0.32	0.00

**Table 4:** Results of the Poisson inverse Gaussian regression model and absolute and relative frequencies of the risk strata of the social deprivation indicator, Igarassu, 2022.

Strata	SDI		Model with 4 Bands				Model with 2 Bands			
	Min	Max	Coeff. ^1^	*p*-Value	N	%	Coeff. ^1^	*p*-Value	N	%
Very low risk (1) ^2^	0.00	0.20	4.51	0.00	12	11.7%	4.68	0.00	82	79.6%
Low risk (2)	0.21	0.34	−0.13	0.68	37	35.9%	-	-	-	-
Medium risk (3)	0.35	0.48	0.45	0.16	33	32.0%	-	-	-	-
High risk (4)	0.51	1.00	0.93	0.01	21	20.4%	0.81	0.00	21	20.4%
Dispersion parameter	-	-	1.66	0.01	-	-	1.82	0.00	-	-

Source: Authors, 2023. ^1^ Parameter estimates given by the regression model for the respective risk stratum/dispersion parameter. ^2^ Risk stratum 1 represents the intercept of the regression model.

## Data Availability

The original contributions presented in this study. Further inquiries can be directed to the corresponding author.
